# Correction to: Epidemiology and evolution of Middle East respiratory syndrome coronavirus, 2012–2020

**DOI:** 10.1186/s40249-021-00898-1

**Published:** 2021-09-06

**Authors:** An-Ran Zhang, Wen-Qiang Shi, Kun Liu, Xin-Lou Li, Ming-Jin Liu, Wen-Hui Zhang, Guo-Ping Zhao, Jin-Jin Chen, Xiao-Ai Zhang, Dong Miao, Wei Ma, Wei Liu, Yang Yang, Li-Qun Fang

**Affiliations:** 1 Department of Epidemiology, School of Public Health, Cheeloo College of Medicine, Shandong University, 44 West Wenhua Road, Jinan, People’s Republic of China; 2 State Key Laboratory of Pathogen and Biosecurity, Beijing Institute of Microbiology and Epidemiology, 20 Dong-Da Street, Fengtai District, Beijing, 100071 People’s Republic of China; 3 Department of Biostatistics, College of Public Health and Health Professions, and Emerging Pathogens Institute, University of Florida, Gainesville, FL USA; 4 Department of Epidemiology, Ministry of Education Key Lab of Hazard Assessment and Control in Special Operational Environment, School of Public Health, Air Force Medical University, Xi’an, People’s Republic of China; 5 Department of Medical Research, Key Laboratory of Environmental Sense Organ Stress and Health of the Ministry of Environmental Protection, PLA Stragetic Support Force Characteristic Medical Center, Beijing, People’s Republic of China; 6 Logistics College of Chinese People’s Armed Police Forces, Tianjin, People’s Republic of China

## Correction to: Infect Dis Poverty (2021) 10:66 10.1186/s40249-021-00853-0

Following publication of the original article [1], it was found that the figure 1 is incorrect. The correct figure (1) is provided in this erratum.

**Fig. 1:**
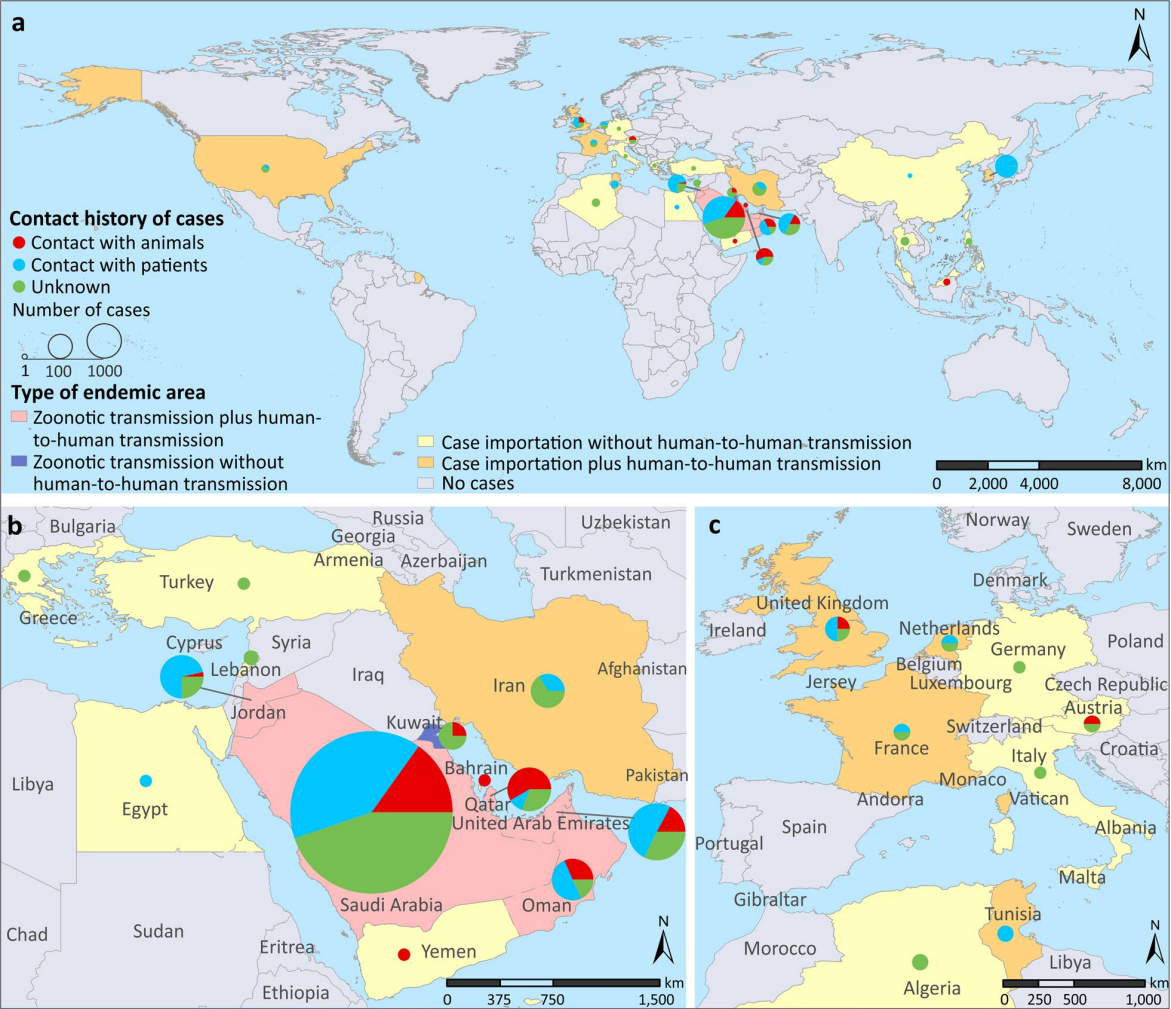
Distribution of human MERS cases in the world **a**, in the Middle East **b** and in Europe **c** during 2012–2020. Countries were colored according to the dominant transmission type: (i) zoonotic transmission plus human-to-human transmission, (ii) zoonotic transmission without human-to-human transmission, (iii) imported infection plus human-to-human transmission, and (iv) imported infection without human-to-human transmission

The original paper has been updated.
